# Magnetic Resonance Imaging of Chiari Malformation Type I in Adult Patients with Dysphagia

**DOI:** 10.1155/2019/7485010

**Published:** 2019-05-14

**Authors:** Feng Lu, Zan Chen, Hao Wu, Feng-Zeng Jian

**Affiliations:** ^1^Department of Neurosurgery, Xuanwu Hospital, Capital Medical University, 45 Changchun St, Beijing 100053, China; ^2^Department of Neurosurgery, South Branch of Fujian Provincial Hospital, 516 South Jinrong Rd, Fuzhou 350001, China

## Abstract

**Objective:**

To explore the magnetic resonance imaging (MRI) characteristics of Chiari malformation type I (CMI) in patients with dysphagia.

**Methods:**

Adult patients diagnosed with CMI were retrospectively and consecutively reviewed from January 2013 to December 2016. Symptoms and medical characteristics were recorded. According to the clinical manifestations, we divided the patients into two groups. The first group had 21 patients with symptoms of dysphagia and the second group had 71 patients with nondysphagia symptoms. Various length or angle measurements of the posterior cranial fossa (PCF), syringomyelia, and degree of cerebellar tonsillar herniation were investigated using magnetic resonance imaging (MRI). Univariate, correlation, and multivariate logistic regression analyses were used to compare and analyze the data of the two groups.

**Results:**

The mean length of the clivus, height of PCF, and slope inclination angle of clivus significantly decreased in the dysphagia group compared to the nondysphagia group. The mean cranial spinal angle (CSA) and degree of cerebellar tonsillar herniation were significantly larger in the dysphagia group. There were no correlations between the age, sex, disease duration, and the length of cerebellar tonsillar herniation or CSA. There was a positive correlation between dysphagia level and CSA (r=-0.50; p=0.021). Among CSA, age, sex, the degree of tonsillar herniation, syringomyelia, and disease duration, CSA was the individual sign that correlated significantly with dysphagia (OR: 1.447; 95% CI: 1.182-1.698; P<0.001). Interactions between CSA and the degree of cerebellar tonsillar herniation, syringomyelia, and dysphagia existed (OR: 1.104; 95% CI: 1.042-1.170; P=0.001 and OR: 1.081; 95% CI: 1.023-1.142; P=0.006, respectively).

**Conclusions:**

The CMI patients with dysphagia were more likely to have a large CSA on MRI compared with CMI patients without dysphagia. An increased probability with syringomyelia or length of cerebellar tonsillar herniation can enhance the contribution of CSA to dysphagia in patients with CMI.

## 1. Introduction

Chiari malformation type I (CMI) is a cerebellar tonsil herniation of the foramen magnum, which can lead to compression of posterior cranial fossa (PCF) contents or pathologic obstruction of fourth ventricle cerebrospinal fluid flow [1, 2]. Clinical manifestations are diverse and include occipital headaches, deep sensitivity disturbances, gait instability, dysphagia, and pyramidal bundle signs [3–6]. Among these symptoms, dysphagia is one of the most important because it can have fatal health consequences and negatively impact patient safety and well-being [7, 8]. Respiratory aspiration is one of the most severe manifestations of dysphagia, which can result in lung infections, undernourishment, prolonged hospital stays, and death [9]. A few reports looking at swallowing dysfunctions in CMI patients exist [10–12], and they were defined as an unexplained and atypical symptom of CMI [13]. However, the pathophysiologic mechanisms underlying dysphagia in CMI patients are still unclear.

T2-weighted magnetic resonance imaging (MRI) scan can show the cranial anatomic and osseous structures so that researchers can complete more accurate morphologic measurements. Therefore, we conducted a retrospective study in a group of CMI patients and explored the MRI characteristics and predictors of dysphagia in CMI patients.

## 2. Materials and Methods

### 2.1. Patients and Clinical Information

Adult (>18 years old) patients diagnosed with CMI were consecutively chosen and retrospectively reviewed from January 2013 to December 2016. CMI was diagnosed based on cerebellar tonsillar herniation of more than 5 mm below the foramen magnum as diagnosed by magnetic resonance imaging (MRI) T1-weighted scans and clinical manifestations [14]. The inclusion criteria were CMI symptoms and a tonsillar herniation of >5 mm. In our practice, we referred patients to a neurologist for clinical assessments before inclusion if a nonspecific headache is reported, which was to exclude causes other than CMI. We excluded patients who were previously diagnosed with swallowing difficulties caused by primary gastroenterological disorders. We also excluded patients with a history of occipital-cervical anomalies, meningitis, intracranial lesions, encephalitis, spine or brain surgeries, head trauma, and atlantoaxial dislocation, which can cause similar symptoms.

Consecutive clinical and radiologic data of 92 symptomatic adult patients (26 males and 66 females) with CMI were retrospectively reviewed from January 2013 to December 2016. Seventy-one patients with symptoms such as cervical neck pain, occipital headache, paresthesia, movement dysfunction, muscle atrophy, and ataxia, but without dysphagia, were classified into the nondysphagia group. Twenty-one patients who had dysphagia with other symptoms were classified into the dysphagia group. All patients in this group underwent the Kubota water-drinking test to evaluate their swallowing function. Specific criteria included the following: (1) Level I: the drinking was finished by swallowing once successfully within 5s without choking; (2) Level II: the drinking was finished by swallowing once successfully within 5s with choking, or by swallowing twice in more than 5s without choking; (3) Level III: the drinking was finished by swallowing once successfully in more than 5s with choking; (4) Level IV: the drinking was finished by swallowing more than twice in more than 5s with choking; (5) Level V: repeated choking with difficulty in swallowing all water within 10 seconds. The criteria of dysphagia were having swallowing function above level III.

Clinical characteristics such as age, sex, height, weight, and medical history were collected.

### 2.2. Radiologic Evaluations

Patient radiologic images were acquired using a 1.5 T MRI unit (Philips InteraAchieva, Philips Medical Systems, Best, The Netherlands). PCF length and angle measurements were manually measured on the mid-sagittal slice of the MRI by the Picture Archiving and Communication System (PACS), which was implemented by three attending radiologists who were blinded to the patients' clinical manifestations [15].

**Figure 1:**
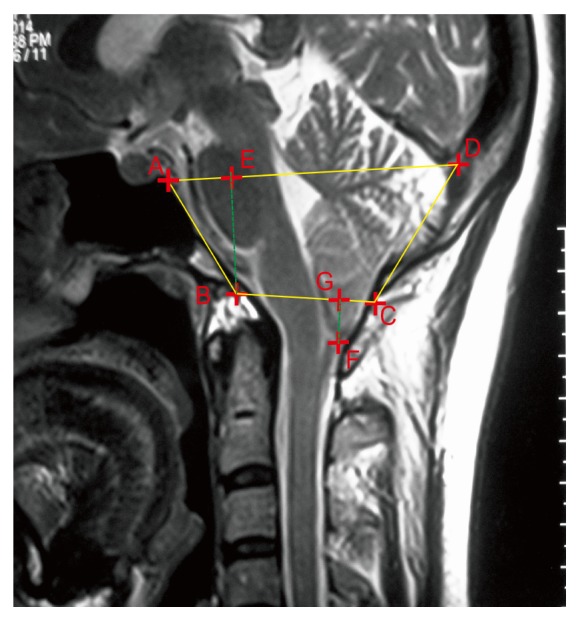
Osseous marks and nerve tissue structure of PCF and liner measurements on the median sagittal MRI scan. A: saddle back, B: the front edge of foramen magnum, C: the back edge of foramen magnum, D: the inside pillow tuberosity, and F: the lower end of the cerebellar tonsils.

**Figure 2:**
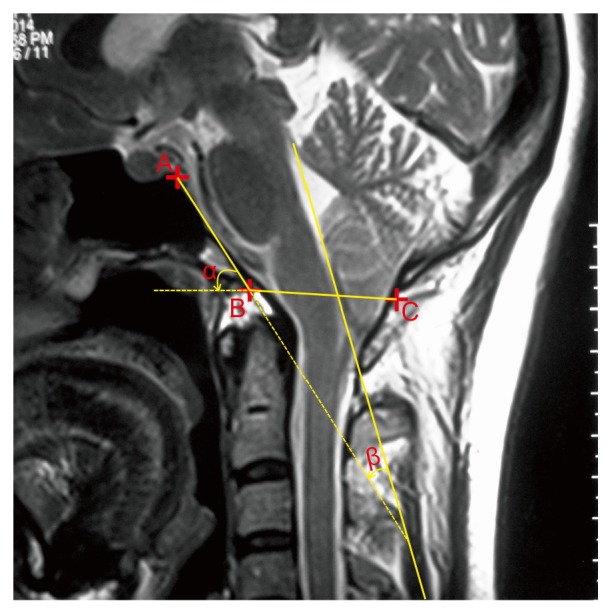
The angle measurements on the median sagittal MRI scan.

PACS was used to obtain linear distance and angle data, such as the length of clivus (AB), anteroposterior diameter of the foramen magnum (BC), supraocciput length (CD), anteroposterior diameter of the PCF (AD), height of the PCF (BE, the vertical distance from point B to the straight line, AD), and degree of cerebellar tonsillar herniation (FG, vertical distance from point F to the straight line, BC) (Figure 1); *α* is slope inclination angle of the clivus (the acute angle between the occipital slope axis and the foramen magnum plane); and *β* is cranial spinal angle (CSA, the acute angle between the extending line of the occipital slope and the extending line of the fourth ventricle floor) (Figure 2). The syringomyelia was evaluated on both the T1- and T2-weighted MRI images.

### 2.3. Statistic Methods

All the data were analyzed using the IBM SPSS Statistics version 19.0 (IBM Corp., Armonk, NY) and presented as the mean ± standard deviation. Pearson's *χ*2 test or Fisher's exact test was used to analyze categorical data, and Student's t-test or the Mann-Whitney U test was used to analyze continuous data. Correlation analyses, including the Pearson correlation for continuous variables and Spearman for categorical variables, were used to evaluate the risk variables. Logistic regression analysis was used to examine the effects of CSA and interactions on symptoms. When statistically significant differences (P<0.05) were found, the odds ratio (OR) and 95% confidence interval (CI) were calculated to further identify the relationships between CSA and dysphagia. A two-sided P value of <0.05 was considered statistically significant.

## 3. Results

Fifty-three patients with syringomyelia were in the nondysphagia group, and 14 patients were in the dysphagia group, with a mean age of 43.9 and 45.7 years, respectively. In the dysphagia group, there were 15 patients with level III and 6 with level IV swallowing function. The length of clivus was significantly decreased in the dysphagia group compared with the nondysphagia group (P<0.01). The mean height of the PCF in the dysphagia group was shorter than that in the nondysphagia group (P<0.05). The mean degree of cerebellar tonsillar herniation in the dysphagia group was larger than that in the nondysphagia group (P<0.01). The mean slope inclination angle of clivus was significantly smaller in the dysphagia group than that in the nondysphagia group (P<0.05). The mean CSA was significantly larger in the dysphagia group than that in the nondysphagia group (P<0.01).

No statistical differences were found in the anteroposterior diameter of the foramen magnum, supraocciput length, and the anteroposterior diameter of the posterior fossa between the two groups. No important differences in the ratio of patients with syringomyelia between the two groups were seen (Table 1).

There were no also correlations between age, sex, disease duration, and degree of cerebellar tonsillar herniation. Similarly, the CSA did not show relevance with age, sex, and disease duration (Table 2). The level of dysphagia in the dysphagia group had a positive correlation with CSA (correlation coefficient=-0.50; p=0.021) (Table 3).

Among the CSA, age, sex, the degree of tonsillar herniation, syringomyelia, and disease duration, CSA was the only sign significantly correlated with dysphagia (OR: 1.447; 95% CI: 1.182–1.698; P<0.001). Interactions between the CSA and the degree of cerebellar tonsillar herniation with syringomyelia and dysphagia were present (OR: 1.104,95% CI: 1.042–1.170; P=0.001; OR: 1.081; 95% CI: 1.023–1.142; P=0.006, respectively) (Table 4).

## 4. Discussion

We present the first retrospective study exploring the magnetic resonance imaging (MRI) characteristics of Chiari malformation type I (CMI) in patients with dysphagia. Dysphagia, associated with lower respiratory tract infections, pneumonia, and other complications, is one of the most severe symptoms among CMI patients. In our study, we found that the mean length of clivus, height of PCF, degree of cerebellar tonsillar herniation, inclined slope of the angle of clivus, and CSA were significantly different between the two groups. The level of dysphagia in the dysphagia group had a positive correlation with CSA (correlation coefficient=-0.50; p=0.021). Among CSA, age, sex, disease duration, degree of tonsillar herniation, and syringomyelia, a large CSA was the most important risk factor for the development of dysphagia in CMI patients (OR: 1.447; 95% CI: 1.182-1.698; P<0.001). The results indicated that CSA could play an important role in dysphagia.

According to our study, CSA was defined as the acute angle between the extending line of the occipital clivus and the extending line of the fourth ventricle floor, which can reflect the tension of the brain stem. The coordinated muscular performance regarding swallowing is controlled by motor and sensory innervations from the cranial nerves that include the trigeminal (V), facial (VII), lower cranial nerves glossopharyngeal (IX), vagus (X), accessory (XI), and hypoglossal (XII) [16]. Swallowing disorders indicate a deficiency in the contraction of the elevator and tensor muscles of the soft palate as in the case of a disorder of cranial nerves IX and X, which may be secondary to elongation lesions [17]. The pharynx receives motor innervations from the pharyngeal branch of the vagus, which consists principally of filaments from the cranial portion of the accessory nerve; the larynx is innervated by the recurrent laryngeal nerves. The simultaneous dysfunction of these nerves is highly indicative of nerve trunk lesions rather than individual branches, as the larynx and pharynx are innervated by vagal branches. Therefore, the bilateral and simultaneous dysfunction of these structures can only result only from an insult to the vagal trunks [18].

Dysphagia has been observed in clinical manifestations of lower cranial nerve paralysis in various conditions [19–21]. It was reported that lower cranial nerve paralysis could be associated with nerve traction, nerve sectioning, and other problems inherent to the disease [21]. Thus, dysphagiain CMI patients could be explained by lower cranial nerves traction owing to the malformation. In our opinion, because cranial nerves arise from the brain stem, patients with larger CSAs can have increased lower cranial nerve tension that could lead to lower cranial nerve damage and paralysis. Sole PCF decompression might not be useful in decreasing nerve traction in some CMI patients, which could lead to severe dysphagia, and, therefore, correcting CSA to decrease nerve traction might be necessary. We consider that CSA could be an important dysphagic symptom predictor in CMI patients.

The interactions between the CSA and the length of cerebellar tonsillar herniation or syringomyelia were also proven in lower cranial nerve paralysis. Significant interactions between the CSA and the degree of tonsillar herniation indicated that increased tonsillar herniation resulted in more patients with dysphagia and larger CSAs in CMI patients (P<0.05). Cerebellar tonsillar herniation could aggravate lower downward cranial nerve traction leading to the aggravation of lower cranial nerve paralysis. In CMI patients, Pollack et al. [18] reported that caudal displacement of the cerebellar tonsils through the foramen magnum could cause lower cranial nerve injury. In some patients, lower cranial nerve paralysis could manifest as dysphagia and be concomitant with syringomyelia. Oldfield et al. [3] reported that cerebellar tonsillar herniation propels cerebral spinal fluid in the spinal subarachnoid space into the interior of the spinal cord and leads to dilatation that can be seen on MRI. Some authors reported that a relationship between syringomyelia and herniation length in CMI exists [22, 23]. Therefore, CSA could be related to syringomyelia. In our series, a patient with CSA had syringomyelia and dysphagia (OR: 1.081; 95% CI: 1.023-1.142; P=0.006), which suggested that the high incidence of syringomyelia was associated with the incidence of dysphagia in patients with larger CSAs but not lower angles. It is difficult to understand why syringomyelia is affected by CSA. However, a possible explanation could be that larger CSAs place cerebral spinal fluid in the front of the medulla that propels the cerebral spinal fluid into the subarachnoid space and interior spinal cord, which leads to syringomyelia, which was similar to Oldfield's report [3].

So larger CSAs cannot be the only predictor of the clinical manifestations of dysphagia on MRI images, especially in the patients with covert dysphagia, but could have some value in choosing surgical procedures. As is already known, the surgical goal in the treatment of symptomatic CMIs is to enlarge the PCF volume and renew CSF flow across the craniocervical junction [22, 23]. According to our results, in some patients with severe dysphagia, the CSA in CMI patients needs to be evaluated and corrected. After PCF decompression, extra occipitocervical correction and fixation might be needed in some patients with severe dysphagia [24].

### 4.1. Study Limitations

This study has several limitations. The main limitations are inherent in the nature of a retrospective study and the small sample size. In addition, the relationship between the larger CSAs and surgical outcomes remains unknown, and further studies are needed.

## 5. Conclusions

According to our research, some CMI patients with severe dysphagia have larger CSAs. The measurement of CSA on MRI may find covert dysphagia in CMI patients. And, patients with syringomyelia or cerebellar tonsillar herniation have an increased likelihood of developing CSAs, which increases the probability of having dysphagia; and therefore, these patients might need decreased traction of the lower cranial nerves. The preliminary findings need to be validated in a larger study.

## Figures and Tables

**Table 1:** Patient clinical characteristics.

	Total (*n*=92)	No dysphagia (*n*=71)	Dysphagia (*n*=21)	*P*
Age (years)	44.3 (10.8)	43.9 (10.2)	45.7 (12.8)	0.498
Men (*n*, %)	26 (28.3)	23 (32.4)	3 (14.3)	0.167
Disease duration (months)	24.00(6.00, 72.00)	24.00(5.00, 72.00)	48.00 (24.00, 120.00)	0.052
BMI (kg/m^2^)	24.54 (3.36)	24.65 (3.45)	24.26 (3.18)	0.656
Clivus (cm)	3.62 (0.44)	3.69 (0.41)	3.35 (0.46)*∗*	0.003
Foramen magnum (cm)	3.26 (0.40)	3.27 (0.33)	3.24(0.57)	0.793
Supraocciput (cm)	4.09 (0.72)	4.10 (0.71)	4.05 (0.75)	0.817
Length of PCF (cm)	7.81 (0.66)	7.84 (0.67)	7.72 (0.63)	0.489
Height of PCF (cm)	2.83 (0.42)	2.90 (0.41)	2.63 (0.40)*∗*	0.013
Tonsillar herniation (cm)	0.97 (0.41)	0.91 (0.36)	1.18 (0.48)*∗*	0.009
Syringomyelia (*n*, % )	67 (77.0)	53 (79.1)	14 (70.0)	0.383
Slope Inclination Angle (°)	45.10 (13.36)	46.77 (12.86)	39.43 (13.77)*∗*	0.026
Cranial Spinal Angle (°)	22.75 (6.73)	20.85 (4.76)	29.15(8.40)*∗∗*	<0.001

Continuous data were shown as mean (SD) and categorical data were *n* (%). Abbreviations: PCF, posterior cranial fossa. ^*∗*^*P*<0.05; ^*∗∗*^*P*<0.001.

**Table 2:** Association of risk variables with tonsillar herniation and cranial spinal angle.

Group		Tonsillar herniation		CSA	
		*r*	*P*	*r*	*P*
Non-dysphagia	Age	-0.120	0.342	-0.083	0.490
	Sex	-0.036	0.775	0.082	0.499
	Disease duration	0.152	0.226	-0.012	0.924

Dysphagia	Age	-0.300	0.199	-0.427	0.053
	Sex	0.012	0.959	0.067	0.772
	Disease duration	-0.210	0.374	0.092	0.691

Abbreviations: CSA: cranial spinal angle. *∗*P<0.05; *∗∗*P<0.001.

**Table 3:** Association of risk variables and dysphagia level in dysphagia group.

variables	Dysphagia level	
	*r*	*P*
CSA	-0.500	0.021
Clivus length	-0.209	0.390
Height of PCF	-0.240	0.323
Slope Inclination Angle	-0.217	0.345

Abbreviations: CSA: cranial spinal angle. *∗*P<0.05; *∗∗*P<0.001.

**Table 4:** Effects of CSA and interactions on dysphagia.

	*β (SE)*	*OR*	*95%CI*	*P*
CSA	0.348 (0.093)	1.447	(1.182-1.698)	0.001
Age (years)	0.044 (0.040)	1.045	(0.697-1.130)	0.269
Men (n, % )	-1.209 (0.930)	0.299	(0.048-1.848)	0.194
Disease duration	0.000 (0.004)	1.000	(0.993-1.007)	0.994
Tonsillar herniation (cm)	1.207 (0.873)	3.343	(0.604-18.514)	0.167
Syringomyelia (*n*, % )	-0.268 (0.822)	0.765	(0.153-3.831)	0.744
CSA by tonsillar herniation	0.099 (0.030)	1.104	(1.042-1.170)	0.001
CSA by syringomyelia	0.078 (0.028)	1.081	(1.023-1.142)	0.006

Abbreviations: CSA: cranial spinal angle. CSA: adjusted by age, sex, degree of tonsillar herniation, syringomyelia, and disease duration.

## Data Availability

The clinical data used to support the findings of this study are included within the article.
